# Impact of COVID-19 on Hospital Admissions for COPD Exacerbation: Lessons for Future Care

**DOI:** 10.3390/medicina58010066

**Published:** 2022-01-01

**Authors:** Michael Lawless, Mark Burgess, Stephen Bourke

**Affiliations:** Northumbria Healthcare Foundation Trust, Cramlington NE23 6NZ, UK; michael.lawless@nhs.net (M.L.); mark.burgess@northumbria-healthcare.nhs.uk (M.B.)

**Keywords:** COPD, SARS-CoV-2, ventilation, oxygen

## Abstract

*Background and Objectives:* Chronic obstructive pulmonary disease (COPD) is a leading cause of death worldwide. Acute exacerbations (AECOPD) are common and often triggered by viral infection. During the COVID-19 pandemic social restrictions, including ‘shielding’ and ‘lockdowns’, were mandated. Multiple, worldwide studies report a reduction in AECOPD admissions during this period. This study aims to assess the effect of the pandemic and Lockdown on the rates of admission with AECOPD and severity of hospitalised exacerbations in the North-East of England. *Materials and Methods:* Data were extracted for patients presenting with a diagnosis of AECOPD or respiratory failure secondary to AECOPD during the ‘COVID-19 period’ (26/3/20–31/12/20) and a date-matched control period from the year previous. We present descriptive statistics and regression analysis of the effects of the COVID-19 period on the rates of hospital admission. *Results:* Compared to the matched control period, the COVID-19 period was associated with fewer AECOPD admissions (COVID-19 = 719, control = 1257; rate ratio 0.57, *p* < 0.001) and shorter length of stay (COVID-19 = 3.9 ± 0.2, control = 4.78 ± 0.2 days; *p* = 0.002), with similar in-hospital plus 30-day post-discharge mortality. Demographics were similar between periods. Only six patients had a positive COVID-19 PCR test. *Conclusion:* During the COVID-19 period there was a substantial reduction in AECOPD admissions, but no increase in overall severity of exacerbations or mortality. Rather than fear driving delayed hospital presentation, physical and behavioural measures taken during this period to limit transmission of COVID-19 are likely to have reduced transmission of other respiratory viruses. This has important implications for control of future AECOPD.

## 1. Introduction

Chronic obstructive pulmonary disease (COPD) is the third leading cause of death worldwide and the sixth highest cause of death in the UK [1,2]. COPD is a preventable and treatable, multifaceted disease influenced by genetic, prenatal/childhood and environmental factors, including tobacco smoke, air pollution and occupational dust and chemicals [3,4]. Patients report persistent, often progressive, respiratory symptoms including breathlessness with reduced exercise tolerance, and chronic cough with variable sputum productivity [5]. An ageing population and continued exposure to risk factors predict that the burden of this disease on health and social care will continue to increase over the next decade [5]. Management strategies for COPD include vaccination against common respiratory pathogens, smoking cessation, pulmonary rehabilitation, and pharmacological measures including inhaled bronchodilators and steroids.

Patients with COPD commonly suffer acute exacerbations (AECOPD), which require hospital admission when severe. AECOPD are not only disruptive to patients [6], but are associated with increased morbidity and mortality [7], and contribute to significant clinical and economic burden worldwide [8,9]. AECOPD are most commonly triggered by viral respiratory infections such as human rhinovirus, respiratory syncytial virus and influenza [10], however may also be caused by bacterial infection and environmental factors such as air pollution. Simple physical interventions have been shown to reduce the risk of respiratory virus transmission in the general population [11].

A quarter of AECOPD are complicated by acute respiratory acidaemia and often require escalation to treatment with non-invasive ventilation (NIV) [12]. Respiratory acidaemia, and chest X-ray consolidation are associated with worse outcome [13]. Of note, UK national audits show a step-change in in-hospital mortality, falling from 7.7–7.8% 2003–2008 to 4.3–3.6% 2014–2019 [14]. This reduction in in-hospital mortality over the last decade may in part be explained by widespread adoption of the BTS oxygen guidelines from 2008 forward with oxygen therapy titrated to target oxygen saturation of 88–92% in most patients [15].

In December 2019 a novel respiratory disease was described in Wuhan, China, caused by the severe acute respiratory syndrome coronovarius-2 (SARS-CoV-2) [16]. The disease termed COVID-19 is associated with a range of symptoms including fever, dyspnoea, dry cough, diarrhoea, and impaired taste/smell sensation, which may progress to lung injury and ARDS [17].

During 2020 worldwide governments instituted policy interventions to reduce COVID-19 transmission. Measures included reinforced hand hygiene, face coverings, social/physical distancing, and full lockdown. In England in 2020 the UK government implemented two major mandatory lockdown periods: Lockdown 1 26/3/20–15/6/20; and Lockdown 2 5/11/20–2/12/20 [18].

Patients with COPD are more likely to develop respiratory complications and have an increased mortality rate secondary to COVID-19 [19,20]. The UK government recommended certain patient groups, including those with COPD, to ‘shield’ or ‘isolate’ during the height of the pandemic to reduce the risk of exposure to COVID-19 infection [21]. Consequently, clinicians adopted more widespread use of telemedicine services using technology to reduce face-face contact and exposure of vulnerable patients. Such services were limited for COPD patients prior to the pandemic; however emergency revisions to COPD NICE guidelines recommended clinicians deliver routine review appointments via remote consultation [22]

Several studies have reported relatively low prevalence of COPD described as a comorbidity in patients admitted to hospital with a primary diagnosis of COVID-19 [19,23]. Furthermore, multiple, worldwide, single-centre studies have reported a reduction in the number of AECOPD admissions [24,25,26]. A recent systematic review of 9 studies from nine countries worldwide reported a 50% reduction in the rate of admission of AECOPD compared with pre-pandemic rates [27]. The reasons for these trends could be behavioural or secondary to the policy interventions implemented during the pandemic resulting in less exposure to triggers for AECOPD.

This study aims to characterise the patient demographic and the effect of Lockdown on the rates and severity of admission with AECOPD in a specialist emergency hospital in the North-East of England.

## 2. Materials and Methods

Consecutive admissions with AECOPD during the COVID-19 pandemic (‘COVID-19 Period’: 26/3/20–31/12/20) and over a date-matched prior period (‘Pre-COVID-19 Period: 26/3/19–31/12/19) were retrospectively captured from coding data at a large general hospital in the Northeast of England, Northumbria Specialist Emergency Care Hospital. The hospital serves over 500,000 patients from urban and rural areas with considerable socioeconomic diversity. Included patients were 35 years and over coded as presenting with a diagnosis code of J44 (capturing exacerbation COPD) or J96 (capturing respiratory failure) with a secondary code of J44. Data were extracted between 26/3/19 and 31/12/20. Date and time points used for analysis, shown in Table 1, were based on the official UK government publication of key COVID-19 pandemic dates [18]. Lockdown 1 and Lockdown 2 were associated with, respectively termed periods ‘Post-Lockdown 1” and “Post-Lockdown 2”.

Key clinical data were captured as described previously in a similar population admitted with AECOPD [28]. Variables captured included age, gender, comorbidity (Charlson Comorbidity index), date of admission and discharge, admission from nursing home (NH) or residential home (RH), length of hospital admission stay in days, COVID-19 swab result at 14 days before and 2 days after admission, readmission within 30 and 90 days, level of care, admission under respiratory consultant, oxygen therapy requirement, presence of consolidation on chest radiograph and NIV therapy requirement. Provision of NIV was verified by our rolling NIV audit, with discrepancies resolved by review of individual case records. The presence of consolidation was confirmed by radiology reports, with review of chest radiographs if unclear. The mortality indicator used in this study is the summary hospital-level mortality indicator (SHMI Mortality), which captures deaths in-hospital plus 30 days post discharge.

Data analysis was performed using SPSS statistics (v27, IBM corp, Armonk, NY, USA). Patient characteristics are presented as mean (SD), median (IQR) and percentage; bivariate comparisons were performed using Student’s t-test, Mann–Whitney U test and Chi squared for parametric, non-parametric and categorical variables. Stepwise logistic regression with backwards elimination techniques were used to analyse the effects of the COVID-19 versus Pre-COVID-19 Period on hospital admission with an exacerbation of COPD. After assessing collinearity, variables associated with admission during the COVID-19 Period (*p* < 0.1) were entered and variables independently associated with this outcome (*p* < 0.05) identified.

## 3. Results

**Figure 1:**
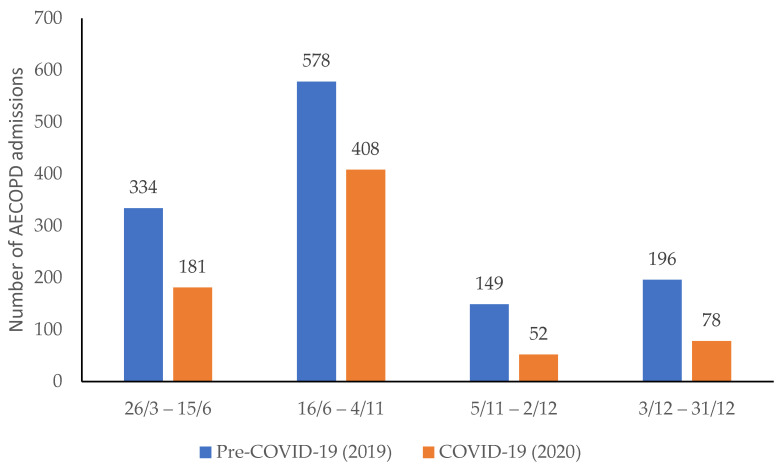
Hospital admission with COPD exacerbation (AECOPD) during COVID-19 period (Orange) compared with pre-COVID-19 period (blue). Numbers of admissions in the period are stated above each bar.

A total number of 1976 admissions with an exacerbation of COPD met the inclusion criteria. The total number of admissions in the COVID-19 period (719) were 57.2% fewer than the date-matched Pre-COVID-19 control period (1257) (*p* < 0.001). Figure 1 shows there were less AECOPD admissions at each of the lockdown and post-lockdown time periods during COVID-19 (orange bars) when compared to the date matched pre-COVID-19 period (blue bars). The length of the different lockdown and post-lockdown periods differed across the year, however when the number of admissions per week (apw) were calculated the respective admission rates were higher in the post-lockdown periods (lockdown 1 v post-lockdown 1 = 16 v 19 apw, lockdown 2 v post-lockdown 2 = 10 v 26 apw). The highest admission rate occurred in December, but admissions across all date range periods were lower during the COVID-19 Period when compared to the previous year. Only six AECOPD patients tested positive for COVID-19 on admission (or within 14 days before and 2 days after admission) during the COVID-19 period, one positive test was confirmed during Lockdown 1, none during Lockdown 2, three during the post-lockdown 1 period and two during the post-lockdown 2 period.

Descriptive statistics for patient characteristics and markers of severity of COPD exacerbation are presented in Table 2. Patient demographics were similar between the two periods. Most patients were female, aged 72(10) and were not nursing/residential home residents. During the COVID-19 period hospital length of stay was significantly shorter, 3.9(5) days versus 4.78(7) days (*p* = 0.002) and there was a trend toward greater comorbidity (*p* = 0.072) in those patients that were admitted. There was no statistically significant difference in in-hospital plus 30-day mortality for those patients admitted with an AECOPD during COVID-19 when compared to the control period (*p* = 0.966). The point estimates of the presence of chest X-ray consolidation and oxygen therapy slightly higher during the COVID-19 Period, but this did not reach statistical significance. Rates of NIV were similar in both periods.

Admission to hospital with AECOPD during the COVID-19 period was used as the dependent variable in a logistic regression with independent variables as listed in Table 3. Odds ratio and *p* values for each variable are shown. A shorter length of stay, greater comorbidity, greater oxygen therapy and less NIV are shown to be associated with admission during COVID-19. The assumptions of uniform variance and linearity for the regression model have all been met.

Next, we explored the effects of Lockdown on AECOPD admission during the COVID-19 period. Descriptive statistics for the patient characteristics and measures of AECOPD severity for the date matched lockdown periods in pre-COVID-19 and COVID-19 periods are shown in Table 4. Patient age, sex and comorbidity were similar during the Lockdown 1 period in comparison to the previous year. These findings were consistent during Lockdown 2. Patients admitted during Lockdown 1 had a significantly greater mortality index and a reduced length of stay in hospital.

In a final set of analysis, we explored the effects of lockdown and the immediate “post-lockdown” period during the COVID-19 Period (Supplementary materials, Table S1) shows descriptive statistics for lockdown and post-lockdown periods. In the Post-Lockdown 1 period more males (48% versus 38%, *p* = 0.026) with increased comorbidity (10.1(9) versus 9.1(10), *p* = 0.022) presented with AECOPD. In the Post-Lockdown 2 period, similarly patients with increased comorbidity presented with an AECOPD (10.2(9) versus 9.0(10), *p* = 0.017), which was associated with an increased finding of consolidation on CXR (26% versus 12%, *p* = 0.049).

## 4. Discussion

This study shows there was an overall reduction in admissions of patients with an acute exacerbation of COPD during COVID-19. This was associated with a shorter length of stay in hospital, however no overall change in the rate of mortality.

Several international studies have shown similar trends of reduced AECOPD admissions during the COVID-19 period [24,26,27]. In addition, we describe a reduction in admissions during both government-enforced Lockdown periods, as well as during the post-lockdown periods when compared to the pre-COVID-19 period. Admission rates only modestly increased during the post-lockdown periods, which suggests patients continued to be cautious thus reducing their exposure to exacerbating factors.

COVID-19 infection has potentially serious consequences for those with respiratory disease, however interestingly, the present study supports other recent reports that there were less hospital admissions with AECOPD during the COVID-19 period [27]. It is possible that the restrictions on social interaction including mask wearing, decreased air pollution, increased smoking cessation, and increased anxiety encouraging greater adherence to medication may have led to a fall in the number of acute exacerbations [29,30,31]. Conversely, these trends may indicate patient behaviour to protect themselves from hospital environments, however if this was the case, then we would have expected more patients to have presented in extremis, which does not seem to be the case. Finally, the reduction in coded exacerbations may in part represent under-diagnosis considering the overlap between some symptoms of COVID-19 and AECOPD [32].

The present study captured hospitalised AECOPD over nine months from the commencement of the first Lockdown period in England (26/3/20) and assessed the impact of both lockdown periods and subsequent easing of restrictions compared to seasonally matched control periods. Other studies investigating the effect of COVID-19 on AECOPD admission captured a shorter period, typically the first 3 months of the pandemic [27]. 

Coronaviruses are known triggers of AECOPD [10], however there has been no definitive evidence that SARS-CoV-2 is a direct trigger of an AECOPD. COPD may be an independent risk factor for severe COVID-19 infection [33], however there are reports that there is indeed a lower prevalence of COPD as a comorbidity in those admitted to hospital with COVID-19 infection [34,35]. The present study saw only six patients with a positive COVID-19 PCR test within 14 days before and 2 days after admission with their AECOPD. Furthermore, only one patient admitted with an AECOPD who tested positive for COVID-19 was admitted during a Lockdown. The other 5 admissions were admitted during either the post-lockdown 1 (3 cases) or post-lockdown 2 period (2 cases). Patients admitted during the “post-lockdown” periods had a higher degree of comorbidity, which itself is a known risk factor for increased hospital admission with COVID-19 [32].

We found that length of hospital stay was shorter during the COVID-19 period compared to the previous year. This effect was more prominent during Lockdown 1, the only period when we saw an increase in mortality. Rather than shorter length of stay reflecting less severe exacerbations occurring during the COVID-19 period, it is likely that this reflects an effect of both patient-led and clinician-led decisions to encourage prompt discharge at a time of fear of nosocomial transmission and high bed pressure. It is reassuring that overall, in-hospital and 30-day post discharge mortality did not increase across the COVID-19 Period (*p* = 0.966). This suggests that patients were indeed safely discharged more readily and not discharged too quickly only to die shortly after. Comparing the subdivided periods during the pandemic to their controls, there may have been a short-lived negative impact initially, with subsequent improvement as patients, clinicians and services adapted. However, caution is required due to the multiple comparisons in subdivided populations.

Chan et al. observed AECOPD in a date matched pre-COVID-19 period and saw no difference in length of stay in hospital [25]. Their study only looked at the first 3 months of the Pandemic in hospitals in Hong Kong, whilst we captured 9 months. There may also have been differences in the behaviour and response of patients, clinicians, and the health services of these two nations. Moreover, Northumbria Healthcare Foundation Trust utilises the DECAF score to risk stratify patients upon admission. Objective clinical risk-stratification may have positively influenced clinician decisions, and our shorter length of stay in hospital may reflect the confidence of the local respiratory physicians in early discharge of such a patient cohort. 

We sought to investigate the effect of Lockdown on the number of AECOPD admissions. We observed reduced rates of admission across the whole COVID-19 period, particularly during both Lockdown periods. In comparison to during Lockdown, admission rate increased during the post-lockdown period, a trend which was greater following Lockdown 2. Patients admitted during the post-lockdown 1 period (summer) were more likely to be male; it is possible that women were more likely to maintain precautions after lockdown was lifted. Patients admitted during the post-lockdown 2 period (winter) were more likely to have increased comorbidity, suggesting, despite a reduction in overall number of exacerbations, that those with poorer baseline health still required hospitalisation. However, normalisation to a COVID-19-era alongside easing of restrictions may have encouraged patients to be more confident to seek medical support. One must be cautious in reading too much into the post-lockdown 2 period, as Tier 4 restrictions were implemented in the North-East from 19 December 2020, with greater restrictions than the post-Lockdown 1 period. 

Overall, our findings are consistent with a true reduction in AECOPD throughout the whole of the COVID-19 period, in particular during lockdown. 

### Study Limitations

The present study shows a reduced rate of admission for AECOPD during COVID-19, a finding reported by several others elsewhere worldwide, however we accept the limitations of generalisability from the study of a single centre in the North-East of England. A key limitation is the reliance on coding data rather than specialist review of all case records; this is mitigated by the consistent approach over both periods, and verification of both the provision of acute NIV (NIV and CPAP share the same code) and presence of consolidation. It would be pertinent to include the admission rates for AECOPD from other Trusts in the area as well as more broadly across the UK to see regional and national trends. Further analysis here may also reveal regional differences in admission and the wider effects of the COVID-19 pandemic on healthcare Trusts. 

Unfortunately, we were unable to access admission DECAF scores and arterial blood gas measurements, which would have provided additional objective data on AECOPD severity. 

Further work directed to investigate the influence of vaccination and more robust treatment strategies for COVID-19 pneumonitis on rates of admission of AECOPD would be an appropriate next step. 

## 5. Conclusions

In conclusion, this study describes reduced hospital admission rates for AECOPD during the COVID-19 pandemic, a trend observed by multiple authors worldwide. For the first time, we describe this trend in association with a shorter in-hospital length of stay, without negative implications on mortality. We suggest that the reduction in hospital admission during COVID-19 was primarily due to a reduction in true severe AECOPD rather than fear of attending healthcare settings, which would have led to more patients presenting in extremis. The reduction in admissions is much greater than that achieved by other strategies and therefore this provides an important means of controlling AECOPD moving forward. 

Whilst we would encourage patients with COPD to lead active lifestyles to prevent deconditioning and to limit social isolation in the interests of wellbeing, we recommend that they do so as safely as possible, and to adopt more stringent precautions during flu season. Precautions they may consider include avoiding contact with people with active respiratory infections, particularly young children; isolation from unwell household members, with increased hand washing, mask wearing and surface disinfection in shared areas; mask wearing on public transport and indoor spaces; choosing to socialize in well-ventilated uncrowded spaces (ideally outside) and avoidance of crowded indoor spaces.

## Figures and Tables

**Table 1:** Dates used for analysis COVID-19 Period versus Pre-COVID-19 Period. Dates based on official UK government publication.

	COVID-19 Period	Pre-COVID-19 Period
Lockdown 1	26/03/20–15/06/20	26/03/19–15/06/19
Post-Lockdown 1	16/06/20–04/11/20	16/06/19–04/11/19
Lockdown 2	05/11/20–02/12/20	05/11/19–02/12/19
Post-Lockdown 2	03/12/20–31/12/20	03/12/19–31/12/19

**Table 2:** Descriptive statistics: pre-COVID-19 period versus COVID-19 period.

	Pre-COVID-19			COVID-19			
	*n*	Mean	SD	*n*	Mean	SD	*p*-Value
Age	1257	72.56	10	719	71.8	11	0.136
Length of stay (days)	1257	4.78	7	719	3.9	5	0.002
Comorbidity	1257	8.96	9	719	9.74	9	0.072
SHMI mortality	1257	0.063	0.04	719	0.063	0.05	0.966
	***n***	**Proportion (%)**		***n***	**Proportion (%)**		***p*-Value**
Gender (male)	540	42.96		319	44.37		0.544
NH or RH resident	92	7.32		50	6.95		0.763
CXR consolidation	147	11.69		103	14.33		0.091
NIV required	179	14.24		89	12.38		0.386
O2 required	315	25.06		201	27.96		0.159

**Table 3:** Results from regression analysis: variables independently associated with admission during COVID-19 Period.

	Odds Ratio	*p*-Value	95% C.I.	
			Lower	Upper
Age	0.99	0.118	0.98	1.00
Male	0.95	0.622	0.79	1.15
Length of Stay (days)	0.97	0.001	0.95	0.99
Comorbidity	1.01	0.015	1.00	1.02
NH or RH resident	0.98	0.914	0.68	1.42
NIV required	0.64	0.015	0.45	0.92
O2 required	1.62	0.001	1.22	2.14
CXR Consolidation	1.27	0.089	0.97	1.67

**Table 4:** Descriptive statistics for Lockdown 1 and 2 in pre-COVID-19 and COVID-19 period.

	Lockdown 1					Lockdown 2				
	Pre-COVID-19		COVID-19			Pre-COVID-19		COVID-19		
	Mean	SD	Mean	SD	*p*-Value	Mean	SD	Mean	SD	*p*-Value
Age	72	10	72	11	0.95	72	9	69	13	0.075
Length of stay (days)	5.34	8	3.67	5	0.003	5.33	7	3.58	5	0.097
Comorbidity	8.57	9	9.12	9	0.52	9.31	9	8.67	9	0.672
SHMI mortality	0.056	0.03	0.068	0.06	0.013	0.066	0.05	0.056	0.04	0.158
	**Count**	**%**	**Count**	**%**	***p*-Value**	**Count**	**%**	**Count**	**%**	***p*-Value**
Gender (male)	147	44	69	38	0.196	68	46	22	42	0.678
NH or RH resident	21	6	9	5	0.543	14	9	2	4	0.203
CXR consolidation	33	10	23	13	0.325	12	8	6	12	0.449
NIV required	52	16	24	13	0.481	21	14	3	6	0.111
O2 required	85	25	51	28	0.503	32	21	12	23	0.810

## Data Availability

Data can be obtained from the authors upon request.

## Supplementary Materials

The following supporting information can be downloaded at: https://www.mdpi.com/article/10.3390/medicina58010066/s1, Table S1: Descriptive statistics comparing lockdown and post lockdown periods during the COVID-19 period.
