# Supplementary material for: Impact of COVID-19 on Hospital Admissions for COPD Exacerbation: Lessons for Future Care
Source: Medicina (Kaunas). 2022 Jan 1;58(1):66. doi: 10.3390/medicina58010066 (PMC8778793; doi:10.3390/medicina58010066)
Supplement: Supplementary file 1 [file medicina-58-00066-s001.zip › medicina-1495184-supplementary.pdf]

medicina MDPI

	Lockdown 1		Post-lockdown 1			Lockdown 2		Post-lockdown 2		
	Mean	SD	Mean	SD	*p*-Value	Mean	SD	Mean	SD	*p*-Value
**Age**	72	11	72	11	0.971	69	11	70	11	0.633
**Length of stay (days)**	3.7	6	4.09	5	0.361	3.8	6	4.1	5	0.911
**Comorbidity**	9.12	10	10.11	9	0.022	9.02	10	10.23	9	0.017
**SHMI mortality**	0.068	0.04	0.059	0.06	0.254	0.056	0.04	0.076	0.06	0.376
	n	%	*n*	%	*p*-Value	*n*	%	*n*	%	*p*-Value
**Gender (male)**	69	38	196	48	0.026	22	42	32	41	0.884
**NH or RH resident**	9	5	7.8	32	0.207	2	4	8.8	7	0.259
**CXR consolidation**	23	13	54	13	0.861	6	12	20	26	0.049
**NIV required**	24	13	56	14	0.879	3	6	6	8	0.672
**O2 required**	51	28	121	30	0.715	12	23	17	22	0.863

Supplementary Table S1. Descriptive statistics comparing lockdown and post lockdown periods during the COVID-19 period.
